# Novel cardiovascular risk markers in women with ischaemic heart disease

**DOI:** 10.5830/CVJA-2014-014

**Published:** 2014-06

**Authors:** Dana Pop, Alexandra Dădârlat, D Zdrenghea

**Affiliations:** University of Medicine and Pharmacy Iuliu Haţieganu, Cluj-Napoca, Romania; University of Medicine and Pharmacy Iuliu Haţieganu, Cluj-Napoca, Romania; University of Medicine and Pharmacy Iuliu Haţieganu, Cluj-Napoca, Romania

**Keywords:** ischaemic heart disease, women, new cardiovascular risk factors

## Abstract

**Abstract:**

The incidence of coronary heart disease in premenopausal women is lower than in men because of their hormonal protection. Angina pectoris occurs in women about 10 years later than in men. However, mortality from ischaemic heart disease remains higher in women than in men. Current studies are focusing on novel cardiovascular risk biomarkers because it seems that traditional cardiovascular risk factors and their assessment scores underestimate the risk in females. Increased plasma levels of these newly established biomarkers of risk have been found to worsen endothelial dysfunction and inflammation, both of which play a key role in the pathogenesis of microvascular angina, which is very common in women. These novel cardiovascular risk markers can be classified into three categories: inflammatory markers, markers of haemostasis, and other biomarkers.

## Abstract

Cardiovascular disease (CVD) represents the leading cause of death among women in Europe. About 53% of female deaths are due to CVD, particularly coronary heart disease and stroke.1-9 The incidence of coronary heart disease is significantly lower in premenopausal women, due to their hormonal protection, but there are reportedly more complex mechanisms involved. Angina pectoris and heart attack occur in women about 10 and 20 years, respectively, later than in men.5

There are significant gender-related differences concerning coronary heart disease. The particularities regarding women are: higher prevalence in women over 75 years, the first coronary event is 10 years later than in men, atypical symptoms, high incidence of non-Q-wave myocardial infarction, and the prevalence of coronary arteries without angiographic findings is twice as common as in men.6

Since 2004, guidelines have been emphasising the importance of recognising cardiovascular risk factors in women and also to classify women at high, intermediate or ‘ideal’ cardiovascular risk.2-4 A high-risk status is given not only by the presence of coronary artery disease, cerebrovascular disease, chronic arterial occlusive disease, aortic aneurysm or a Framingham score over 10%, but also by the presence of chronic kidney disease or diabetes.2

Women who face the threat of cardiovascular disease present with one or more risk factors including: smoking, pro-atherogenic diet, obesity (especially central obesity), family history of cardiovascular disease at a young age, hypertension and dyslipidaemia. Furthermore, it seems that subclinical vascular disease (such as coronary calcification), the metabolic syndrome, a low effort capacity or an abnormal heart rate recovery after the exercise stress test creates a prominent cardiovascular risk among women.2 Latest studies show that women diagnosed with collagen disease (auto-immune disease), a history of pre-eclampsia, gestational diabetes or pregnancy-induced hypertension require strict medical management due to their high predictive ability for the development of cardiovascular disease.2

Ideal cardiovascular health status is gained by women with blood pressure below 120/80 mmHg, total cholesterol level below 200 mg/dl, fasting plasma glucose below 100 mg/dl (without specific treatment), body mass index (BMI) below 25 kg/m^2^ and, undoubtedly, by those who practice intense physical exercise at least 150 minutes per week, or moderate exercise for 75 minutes per week, and by non-smoking women.2

Review of the evidence reveals that compilation of traditional risk factors and cardiovascular risk scores underestimates the risk in women. Therefore, ongoing areas of research are focusing on novel markers of cardiovascular risk. These novel cardiovascular risk biomarkers have been selected because their increased plasma levels worsen endothelial dysfunction and inflammation, both being key players in the pathogenesis of microvascular angina, which is a common phenomenon in women.1

The Women’s Health Initiative hormone trials showed that at least 18 new biomarkers are useful in estimating cardiovascular risk in postmenopausal women. These are lipoprotein (a), homocysteine, insulin, C-reactive protein (CRP), E-selectin, interleukin-6, matrix metalloproteinase-9, fibrin D-dimer, factor VIII, plasminogen activator inhibitor-1 antigen, prothrombin fragment 1.2, plasmin–antiplasmin complex, thrombin-activatable fibrinolysis inhibitor, von Willebrand factor, fibrinogen, haematocrit, leukocyte and platelet counts.10 These novel biomarkers of cardiovascular risk are classified into three categories: inflammatory markers, haemostasis markers, and other biomarkers.

## Inflammatory markers

## High-sensitivity C-reactive protein (hs-CRP)

The latest European guidelines on CVD prevention in clinical practice (2012) recommend the determination of high-sensitivity CRP levels as part of the refined risk assessment in patients with an unusual or moderate CVD risk profile (class IIB, level B).11 Normal values for this inflammatory factor are below 2 mg/dl.

CRP levels in women are higher than in men, especially during puberty.2 The JUPITER trial reported that an hs-CRP value over 2 mg/dl in association with a low-density lipoprotein (LDL) cholesterol value below 130 mg/dl in women without cardiovascular pathology increases the risk of cardiovascular events.12 Moreover, high levels of CRP in women without cardiovascular disease are important predictors for the development of fatal heart attack and stroke.13

The greater the number of cardiovascular risk factors that apply to a woman, the higher her hs-CRP level.13 Elevated CRP levels have been associated with the presence of the metabolic syndrome, diabetes and chronic heart failure. Furthermore, recent studies show that a high CRP value is correlated with an increased incidence and prevalence of auto-immune diseases in women, such as rheumatoid arthritis and lupus erythematosus.3-6

The Women’s Health study demonstrated that the addition of hs-CRP to the Framingham score improved the predictive accuracy of cardiovascular risk, especially in women with a 5–20% risk in 10 years.14 Evidence from the Women’s Ischemia Syndrome Evaluation, a prospective study, reported that high levels of amyloid serum A, IL-6, sICAM1 and CRP had the highest predictive accuracy in 27 347 postmenopausal women apparently without cardiovascular disease.15 The guidelines do not however recommend routine evaluation of this inflammatory biomarker, CRP.2,11

## Fibrinogen

High levels of fibrinogen are associated with an increased risk of cardiovascular disease in both men and women, but there are still substantial gender-specific differences.6,13 On one hand, plasma fibrinogen levels increase with menopause, but also during the use of oral contraceptives and pregnancy.16,17 On the other hand, hormone replacement therapy lowers serum levels of fibrinogen.16

The latest European guidelines on cardiovascular disease prevention in clinical practice recommend the determination of fibrinogen levels as part of a refined risk assessment in patients with an unusual or moderate CVD risk profile (class IIB, level B).11

## Interleukin-6 (IL-6)

IL-6 stimulates hepatic release of CRP and fibrinogen, both acute-phase reactants involved in the process of atherosclerosis and atherothrombosis. Unfortunately, there are contradictory data regarding the role of IL-6 in the development of coronary heart disease in women.10,17

*Atherosclerosis* reported from the British Women’s Heart and Health study that the level of this cytokine was not directly associated with the risk of coronary heart disease.18 Interestingly, the Women’s Health Initiative showed a direct correlation between high levels of IL-6 and ischaemic heart disease.10 Undoubtedly, cardiovascular risk was not assessed only by measuring the IL-6 plasma levels, but also by determining other cardiovascular risk factors.10

## Matrix metalloproteinase-9 (MMP-9)

MMP-9, along with CRP, IL-6 and increased levels of leukocytes may provide accuracy in the prediction of developing coronary heart disease in women.10,17,19

## E-selectin

Various studies impugn the relationship between E-selectin and cardiovascular risk.17,19 On the other hand, there is evidence to support the predictive value of E-selectin for cardiovascular events.17,19,20

## Haemostasis markers

There are sufficient data concerning the association of D-dimer, coagulation factor VII, von Willebrand factor and fibrinogen levels with the risk of coronary heart disease in women (after statistical adjustments for traditional risk factors).21 Studies demonstrated the presence of high levels of coagulation factor VII in women suffering from angina or other cardiovascular diseases.22-25 However, the most eloquent reports support the use of D-dimer in estimating prognosis of cardiovascular death and other events in women.26

## Plasminogen activator inhibitor-1 (PAI-1)

Recent studies identified lower PAI-1 levels in premenopausal than postmenopausal women.16,17 The concentration of PAI-1 was lower in women taking hormone replacement therapy, compared with non-users.6,16

Gene-specific differences and changes in PAI-1 values during the postmenopausal years may be related to PAI-1 gene polymorphism. The 4G/5G mutation was found more frequently among postmenopausal women with coronary heart disease than in premenopausal women.16

## Lipoprotein (a) [Lp(a)]

As is well known, elevated levels of Lp(a) increase the risk of ischaemic heart disease in both men and women. Investigators demonstrated a clear association between Lp(a), LDL cholesterol, hypertension, hyperhomocysteinaemia and hyperfibrinogenaemia in men. Also in women an increase in Lp(a) levels with age has been reported.6

Notably, Lp(a) is an emerging cardiovascular risk factor in both pre- and postmenopausal women as it contributes to the formation of atherosclerosis. Sometimes high levels of Lp(a) are correlated with high CRP levels.27

High Lp(a) values together with abnormal blood lipid levels are risk factors for cardiovascular disease in women, even in those under 60 years.16 New research on women offers strong evidence that heart attack risk increased as Lp(a) levels rose.28

## Lipoprotein-associated phospholipase A2 (Lp-PLA2)

Recent data confirm the involvement of Lp-PLA2 in the development of atherosclerosis by modifying the affinity of LDL particles for extracellular matrix proteins.30-32 Moreover, Lp-PLA2 favours lipid accumulation in arterial walls, lipid peroxidation, and hydrolysis of lysophospholipids and free fatty acids.33,34 Lp-PLA2 may be identified as an independent risk factor for rupture of atheroma plaque and thrombo-embolic events.12

The latest European guidelines on cardiovascular disease prevention in clinical practice recommend the determination of Lp-PLA2 values as part of a refined risk assessment in patients at high risk of a recurrent acute atherothrombotic event (class IIB, level B).11 The 2010 ACCF/AHA Guideline for the Assessment of Cardiovascular Risk in Asymptomatic Adults reported that calculation of Lp-PLA2 levels may be reasonable for cardiovascular risk assessment in intermediate-risk asymptomatic adults (class IIb level B).35

The recent Nurses’ Health study showed that levels of Lp-PLA2 were significantly associated with the incidence of ischaemic heart disease in women.36 According to some research results, women have higher levels of secretory phospholipase A2 (sPLA2) than men. It was reported that elevated sPLA2 levels were correlated with high CRP levels.27,37

## Homocysteine

In general, women present with lower homocysteine values than men, but elevation occurs during the menopausal years.16,38 Also, a number of studies suggested a relationship between serum homocysteine levels and the presence of coronary artery disease in women, but not in men.14 Therefore, it represents a stronger atherogenic factor in women than in men.16 Other studies however did not identify homocysteine as a significant factor in predicting statistical risk of coronary heart disease after adjustment for traditional risk factors, even though they found a positive correlation between this biomarker and ischaemic heart disease.38

Nevertheless, the latest European guideline on cardiovascular disease prevention in clinical practice states that homocysteine may be measured as part of a refined risk assessment in patients with an unusual or moderate CVD risk profile (class IIB, level B).11 The measurement of serum homocysteine levels is not part of the routine screening process for cardiovascular risk assessment.11

## Other markers

## Natriuretic peptides

The Framingham Offspring study showed that 10 elevated biomarkers, and high B-type natriuretic peptides (BNP) indicated cardiovascular risk.39 On the other hand, the Swedish Malmö diet and cancer cohort showed that only BNP and mid-region pro-adenomedulin levels were associated with a doubled cardiovascular risk.40

The 2012 ESC guidelines for the management of heart failure revealed that BNP, N-terminal pro B-type natriuretic peptide (NT-proBNP) and mid-regional pro-atrial natriuretic peptide (MR-proANP) levels showed usefulness in detecting heart failure patients, a differential diagnosis of dyspneoa and risk stratification.41

The KORA study included 1 005 women and men aged between 25 and 75 years. The goal of this study was to determine the variation in the NT-proBNP and BNP levels in a 10-year period. They reported a strong correlation between gender, age and plasma levels of natriuretic peptides. Both NT-proBNP and BNP serum concentrations recorded an elevation during the follow-up period, especially in women.42 However, it has been shown that a BNP value that exceeds 500 pg/ml represents a stronger predictor of death in women than men with heart failure.43

Growth differentiation factor-15 (GDF-15) is a novel biomarker under investigation, which is synthesised in ischaemic myocytes. There is evidence that it strongly indicates an increased risk of cardiovascular death.44

## Conclusion

Despite the use of these novel cardiovascular risk factors, the presence of hypertension, diabetes, physical inactivity and inflammatory markers remain the most potent cardiovascular risk factors in women, regardless of age. Novel cardiovascular risk factors may play a decisive role in the early diagnosis of ischaemic heart disease, especially in women with suspected myocardial ischaemia, but without electrocardiographic, echocardiographic or angiographic findings. However, their routine measurement is difficult to implement. The guidelines regarding coronary artery disease in women could suggest the determination/evaluation of these novel cardiovascular risk factors when a differential diagnosis should be considered.
